# Hidden amorphous phase and reentrant supercooled liquid in Pd-Ni-P metallic glasses

**DOI:** 10.1038/ncomms14679

**Published:** 2017-03-17

**Authors:** S. Lan, Y. Ren, X. Y. Wei, B. Wang, E. P. Gilbert, T. Shibayama, S. Watanabe, M. Ohnuma, X. -L. Wang

**Affiliations:** 1 Herbert Gleiter Institute of Nanoscience, Nanjing University of Science and Technology, 200 Xiaolingwei Avenue, Nanjing 210094, China; 2 Department of Physics and Materials Science, City University of Hong Kong, 83 Tat Chee Avenue, Hong Kong, China; 3 X-ray Science Division, Argonne National Laboratory, Argonne, Illinois 60439, USA; 4 Australian Centre for Neutron Scattering, Australian Nuclear Science and Technology Organization (ANSTO), Locked Bag 2001, Kirrawee DC NSW 2232, Australia; 5 Laboratory of Quantum Beam System, Division of Quantum Beam Engineering, Faculty of Engineering, Hokkaido University, Kita 13, Nishi 8, Sapporo 060-8628, Japan; 6 City University of Hong Kong Shenzhen Research Institute, 8 Yuexing 1st Road, Shenzhen Hi-Tech Industrial Park, Shenzhen 518057, China; 7 Center for Advanced Structural Materials, City University of Hong Kong, 83 Tat Chee Avenue, Hong Kong, China

## Abstract

An anomaly in differential scanning calorimetry has been reported in a number of metallic glass materials in which a broad exothermal peak was observed between the glass and crystallization temperatures. The mystery surrounding this calorimetric anomaly is epitomized by four decades long studies of Pd-Ni-P metallic glasses, arguably the best glass-forming alloys. Here we show, using a suite of *in situ* experimental techniques, that Pd-Ni-P alloys have a hidden amorphous phase in the supercooled liquid region. The anomalous exothermal peak is the consequence of a polyamorphous phase transition between two supercooled liquids, involving a change in the packing of atomic clusters over medium-range length scales as large as 18 Å. With further temperature increase, the alloy reenters the supercooled liquid phase, which forms the room-temperature glass phase on quenching. The outcome of this study raises a possibility to manipulate the structure and hence the stability of metallic glasses through heat treatment.

## Abstract (web summary)

An anomalous exothermal calorimetric peak far below crystallization temperatures in prototypical Pd-Ni-P glasses has been recognized for four decades. Here authors use neutron and high-energy X-ray diffraction to find evidence for a polyamorphous phase transition where medium-range order undergoes large changes while short-range order changes little.

Pd-Ni-P is an excellent glass-forming system, for which centimetre-sized metallic glasses can be made at a cooling rate as slow as 0.17 K s^−1^ (refs 1, 2, 3). In 1976, Chen^4^ observed a broad exothermic peak, which is far below the crystallization temperatures in the differential scanning calorimetry (DSC) curves of (Pd_0.5_Ni_0.5_)_100−*x*_P_*x*_ (*x*=17∼19) amorphous alloys. He proposed that this anomalous exothermal peak, hereafter abbreviated as AEP, corresponds to crystallization catalysed by amorphous phase separation^4^. However, *in situ* small-angle X-ray scattering experiments by Yavari *et al*.^5^ could not confirm the development of compositional heterogeneities in the temperature range of interest. Subsequent study using atom probe tomography^6^ and element-specific transmission electron microscopy (TEM)^7^ also failed to provide evidence of compositional or microstructure modulations before the onset of crystallization. Together, these findings appear to rule out the possibility of nanoscale phase separation. Instead, changes in short-range ordering were suggested as a possible cause for the observed AEP. Indeed, there were hints in early TEM work^8^, which suggested that phase separation was taking place, but at the atomic scale instead of the nanoscale as thought previously.

A similar DSC anomaly has also been reported in a number of other metallic glass alloys, including Zr-Be-X (X=Ti, Nb)^9^,^10^,^11^,^12^, Zr-Ti-Cu-Ni-Be^13^,^14^, Gd-Zr-Al-Ni^15^, Ni-Zr-Nb-Al-Ta^16^, Cu-Zr-Al-Y^17^, Mg-Cu-Ag-Gd^18^,^19^, Fe-M-Y-B (M=Mo, W, Nb)^20^,^21^,^22^, (Fe_0.9_Co_0.1_)_67.5_Nb_4_Gd_3.5_B_25_ (ref. 23), (Fe_0.75−*x*_Dy_*x*_B_0.2_Si_0.05_)_96_Nb_4_ (ref. 24) and (Fe_0.76−*x*_Dy_*x*_B_0.24_)_96_Nb_4_ (*x*=0–0.07)^25^. Many of these alloys have good glass-forming abilities. Microscopic studies were carried out to investigate a scenario of amorphous phase separation before crystallization, but the results are mostly inconclusive^10^,^23^,^26^, due to, for example, possible artefacts arising from sample preparation^27^. *In situ* studies are therefore essential to determine the structural origin of the DSC anomaly.

Here, we employ *in situ* high-energy synchrotron X-ray diffraction, and small-angle neutron scattering (SANS) with simultaneous DSC. Both are bulk techniques; consequently, sample preparation is not an issue and the data interpretation is straightforward. The scattering results were further checked and reconciled with TEM analysis. Our data for Pd-Ni-P glass alloys showed a change that is in perfect correlation with the DSC data, revealing a hidden amorphous phase, the associated polyamorphous or liquid-to-liquid phase transition (LLPT) and a reentrant behaviour^28^ in the supercooled liquid region at higher temperatures. Although polyamorphous phase transitions have been extensively studied, the experimental evidence has been rare and sometimes controversial. Evidence for LLPT has been reported in triphenyl phosphite^29^, confined water^30^ and in metallic glasses close to the melting temperature^31^,^32^,^33^. Polyamorous transition has been previously observed in metallic glasses as well, but under pressure^34^. Nevertheless, the origin of polyamorphous phase transition is far from clear, and has been a subject of intense debate. Our studies of Pd-Ni-P alloys show that at the transition temperature, the underlying atomic structure undergoes significant changes over the medium-range length scale, while the short-range order is little changed.

## Results

### The anomalous exothermal peak

**Figure 1:**
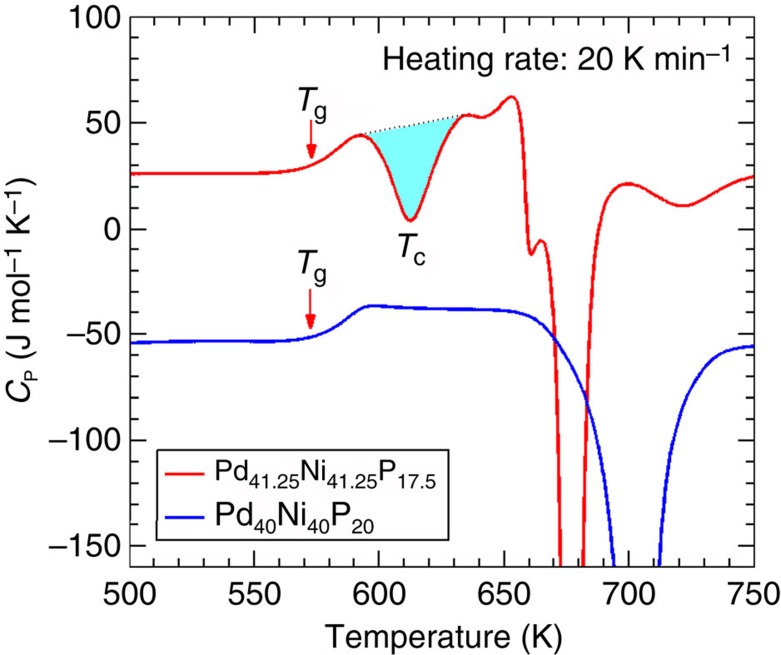
Specific heat (*C*_P_) for as-cast BMGs on heating. For the Pd_41.25_Ni_41.25_P_17.5_ BMG, there is an anomalous exothermal peak at *T*_C_∼612 K, highlighted by the shadow, which is ∼41 K below the crystallization peak at∼653 K. The *C*_P_ data for Pd_40_Ni_40_P_20_, downward shifted by 80 J mol^−1^ K^−1^, are characteristic of a normal BMG, marked by a *T*_g_ and a crystallization peak; there is no DSC anomaly before crystallization. For both samples, the *T*_g_∼570 K.

Figure 1 shows the specific heat capacity, *C*_p_, for two bulk metallic glass (BMG) samples, Pd_41.25_Ni_41.25_P_17.5_ and Pd_40_Ni_40_P_20_. The heating rate was 20 K min^−1^. The DSC scan of Pd_41.25_Ni_41.25_P_17.5_ exhibits an AEP at *T*_C_∼612 K, which is ∼41 K below the crystallization temperature, *T*_*x*_, ∼653 K. The integral area for the AEP is 10.6±0.1 J g^−1^. This value can be compared to the total integral area of the crystallization peak which is 101.4±0.1 J g^−1^. The large exothermal peak at *T*_C_ is strongly indicative of a thermodynamic phase transition. Notice that the *C*_p_ for Pd_41.25_Ni_41.25_P_17.5_ approaches zero at *T*_C_. Thus, if the AEP is indeed due to a phase transition, the associated phases must have comparable potential energies at ∼*T*_C_.

### *In situ* synchrotron diffraction

**Figure 2:**
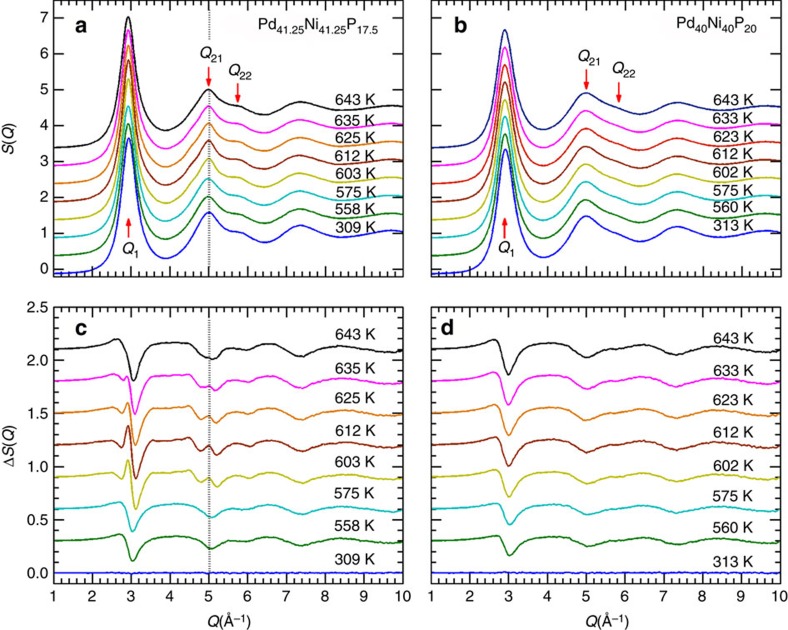
*In situ* X-ray diffraction data. Evolution of synchrotron X-ray diffraction patterns for (**a**) Pd_41.25_Ni_41.25_P_17.5_ and (**b**) Pd_40_Ni_40_P_20_ BMGs during heating at a constant rate of 20 K min^−1^. The structure factor is characterized by a first sharp diffraction peak *Q*_1_, followed by the second diffraction maxima *Q*_21_ and a shoulder *Q*_22_. (**c**,**d**) are the respective difference plots obtained by subtracting the diffraction pattern at *T*=303 K.

Figure 2a,b show the evolution of X-ray structure factor, *S(Q)*, for Pd_41.25_Ni_41.25_P_17.5_ and Pd_40_Ni_40_P_20_ alloys, obtained by *in situ* high-energy synchrotron X-ray measurements, on heating the samples from room temperature to ∼643 K. To detect the subtle structural changes during heating, the *S(Q)* at *T*=303 K was subtracted, and the difference curves, Δ*S(Q)*, are plotted in Fig. 2c,d, respectively. For Pd_40_Ni_40_P_20_, the difference curves display a normal change with increasing temperature, in which the diffraction peaks shift to lower *Q* values accompanied by peak broadening and a decline in peak intensity. For Pd_41.25_Ni_41.25_P_17.5_, however, unusual changes can be readily identified in the vicinity of *T*_C_. For example, on heating, the *Q*_21_ peak initially broadens, but above *T*_g_∼570 K, *Q*_21_ begins to sharpen until the temperature reaches *T*_C_∼612 K, and then resumes broadening at higher temperatures. This is evidenced by the development and disappearance of an extra peak at the position of *Q*_21_ in the Δ*S(Q)* curves (Fig. 2c). These anomalous structure changes point to a phase transition in Pd_41.25_Ni_41.25_P_17.5_, and that the new phase, hereafter referred to SCL2, is metastable.

**Figure 3:**
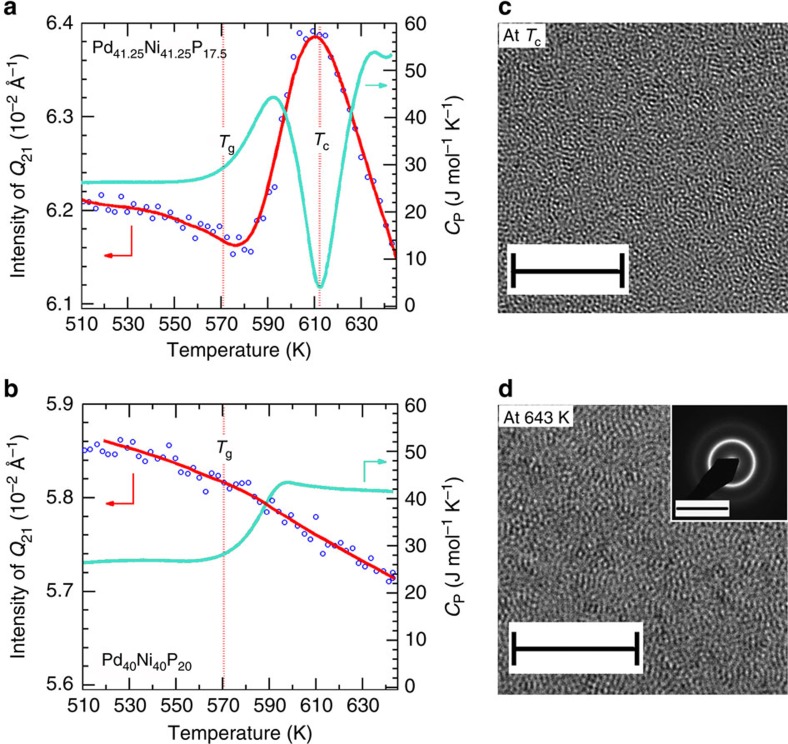
Evidence for the hidden amorphous phase. Temperature dependence of the *Q*_21_ peak intensity (integrated over a *Q* range of 4.98–5.00 Å^−1^ ) versus *C*_P_ for (**a**) Pd_41.25_Ni_41.25_P_17.5_ and (**b**) Pd_40_Ni_40_P_20_ BMGs. The *T*_g_ and *T*_C_ are marked by the vertical dashed lines. The solid lines are the results of a smooth spline fitting, which serve as a guide to the eyes. (**c**) Double Cs-corrected TEM image of Pd_41.25_Ni_41.25_P_17.5_ on a sample quenched at *T*_C_; scale bar, 5 nm. (**d**) High-resolution TEM of Pd_41.25_Ni_41.25_P_17.5_ on a sample quenched at 643 K, after the transition has ended; scale bar, 5 nm. The inset shows the SAED pattern; scale bar, 10 nm^−1^.

To correlate the observed anomalies in structure and thermodynamics, the evolution of the *Q*_21_ peak intensity (integrated over a *Q* range of 4.98–5.00 Å^−1^) and the *C*_p_ data are plotted together in Fig. 3. For Pd_41.25_Ni_41.25_P_17.5_, the intensity of *Q*_21_ shows a rise and fall through *T*_C_, which correlates perfectly with the changes seen in *C*_p_. For Pd_40_Ni_40_P_20_, on the other hand, the intensity for *Q*_21_ shows a continuous decline with temperature, except for a point of inflection at *T*_g_. Subtle changes at other *Q* values can also be seen, showing similar temperature dependence. An example is shown for peak *Q*_1_ in Supplementary Fig. 1. The position of *Q*_1_ shows consistent changes, see Supplementary Fig. 2 and Supplementary Note 1.

### The amorphous hidden phase and reentrant behaviour

Note that no crystalline peaks were detected in the X-ray diffraction patterns, even in the Δ*S(Q)* plots, which are sensitive to structure changes as low as ∼10^−6^ volume fraction transformed. High-resolution TEM was employed to confirm the amorphous nature of the new phase in the vicinity of *T*_C_. Figure 3c shows a double Cs-corrected TEM image taken on a Pd_41.25_Ni_41.25_P_17.5_ BMG, quenched from *T*_C_∼612 K. The TEM image is typical of an amorphous alloy: uniform and maze-like. This result confirmed that the Pd_41.25_Ni_41.25_P_17.5_ sample remained in an amorphous state at *T*_C_.

At *T*>*T*_C_, the extra intensities in the Δ*S(Q)* curves diminished with increasing temperature and disappeared entirely at *T*=643 K. At this point the *S(Q)* reverted back to the shape at *T*=575 K. A high-resolution TEM was taken on a Pd_41.25_Ni_41.25_P_17.5_ sample quenched from 643 K, after the transformation at *T*_C_ ended; again, an amorphous structure is observed. The inset of Fig. 3d shows the selected area electron diffraction (SAED) pattern, further confirming the amorphous nature of the alloy at this temperature. These observations suggest that at *T*>*T*_C_, after the SCL2 phase dissolves, the Pd_41.25_Ni_41.25_P_17.5_ BMG reenters the supercooled liquid of the room-temperature glass phase.

### Simultaneous SANS-DSC

**Figure 4:**
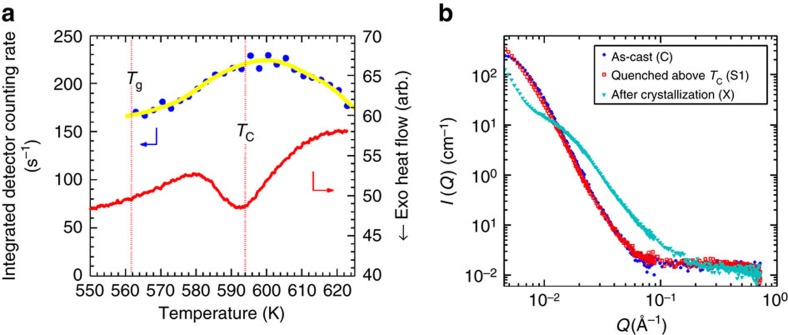
Simultaneous SANS-DSC data for Pd_41.25_Ni_41.25_P_17.5_. (**a**) The integrated detector counting rates as a function of temperature. The simultaneous DSC scan is superimposed, which shows a *T*_C_∼594 K at a heating rate of 2.5 K min^−1^. The coexistence of two phases can be clearly seen in the vicinity of *T*_C_. (**b**) Full-*Q* range SANS data for three samples: as-cast condition (C), quenched from 623 K after the transformation had ended (S1), and quenched after crystallization at 673 K (X). The nearly complete overlap of the SANS profiles between the as-cast and S1 samples demonstrates the reentrant behaviour.

SANS measurements, which are sensitive to composition or density fluctuations on the nanometer scale, provided further evidence of a reentrant supercooled liquid phase at *T*>*T*_C_. The Pd_41.25_Ni_41.25_P_17.5_ sample was heated, at a heating rate of 2.5 K min^−1^, in a DSC furnace installed on the SANS instrument. SANS data were recorded *in situ* over a *Q* range of 0.0065–0.1000 Å^−1^. The integrated SANS detector count rate is plotted in Fig. 4a, along with the DSC data. The AEP is readily identified in the simultaneous DSC data, although the peak temperature *T*_C_ is slightly lower due to the slower heating rate. The SANS intensity shows a rise and fall through *T*_C_, once again demonstrating the development and disappearance of the SCL2 phase as the BMG sample proceeded through the phase transition. At *T*=623 K, the SANS intensity dropped to the level at *T*_g_, showing that the metastable SCL2 phase had dissolved and the phase transition was complete. The sample was then quenched to room temperature to retain its phase at *T*=623 K (S1). Full *Q* range (0.003–0.727 Å^−1^) SANS data were collected and compared with that of an as-cast sample (C) (see Fig. 4b). Their SANS profiles almost overlapped completely with each other, indicating that samples C and S1 are identical, from both chemistry and density perspectives. This observation reinforces our conclusion that after the SCL2 phase dissolves, the Pd_41.25_Ni_41.25_P_17.5_ sample indeed reenters the high-temperature supercooled liquid phase, which on quenching forms the room-temperature glass phase. In comparison, a crystallized sample was also measured and the results were superimposed in Fig. 4b. The crystallized sample shows significant SANS intensity over a *Q* range of 0.002–0.02 Å^−1^. At lower *Q* values, the SANS intensity is lower, suggesting some growth of the nanoscale inhomogeneities in the as-cast samples at high temperatures when the sample crystallized.

### Structure analysis in real space

**Figure 5:**
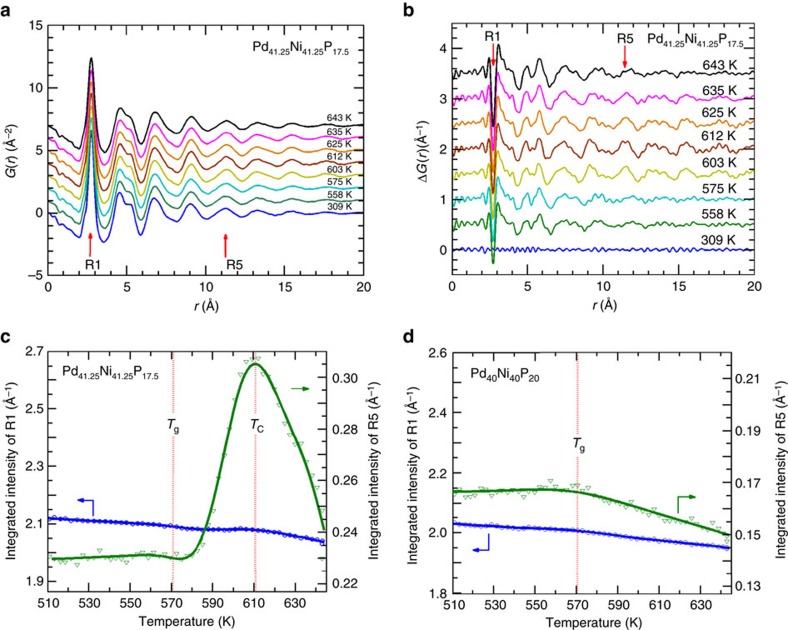
Structural analysis in real space. All data are for sample Pd_41.25_Ni_41.25_P_17.5_, unless otherwise noted. (**a**) Reduced pair-distribution function *G(r)*. (**b**) Differential *G(r)* obtained by subtracting the data set at *T*=303 K. (**c**) Integrated intensity of *G(r)* over the first (R1) and fifth (R5) shell (for regions where *G(r)*≥0). (**d**) Same as (**c**) but for Pd_40_Ni_40_P_20_.

Having established the hidden amorphous and the reentrant behaviour, we turn to real-space structure analysis to obtain atomistic insights of the structure evolution through the transition. The pair-distribution functions were obtained by Fourier transform of the structure factors. The reduced pair-distribution function, *G(r)*, is presented in Fig. 5a for selected temperatures through *T*_C_. Clear changes can be seen in the differential *G(r)* obtained by subtracting the reference data set at *T*=303 K (see Fig. 5b). The first shell, R1 or the short-range order, shows a small change through the transition. Much more pronounced changes are seen in higher-order shells. For example, the intensity of the fifth shell, R5, clearly shows a rise and fall with increasing temperature. In Fig. 5c, the integrated intensity of *G(r)* over the first and fifth shells are plotted for comparison (over comparable ranges, that is, ∼±20% of the initial values). The intensity of R5 increases dramatically as *T*_C_ is approached, and starts to decrease at *T*>*T*_C_, and then returns to the level before AEP. The intensity of R1 shows similar, but much more subdued, temperature dependence. These results demonstrate that the phase transition through *T*_C_ is driven by the medium-range order; the short-range order is hardly changing. As a comparison, the temperature dependence of the *G(r)* intensity for Pd_40_Ni_40_P_20_ BMGs is shown in Fig. 5d. In metallic glasses, the first shell or short-range order is characterized by solute-centred clusters, while the higher-order shells describe the packing or connectivity of the clusters. Thus, the change of *G(r)* in the higher-order shells (for Pd_41.25_Ni_41.25_P_17.5_ alloys), which can be seen at a distance as large as 18 Å in Fig. 5b, indicates a rearrangement of the atomic clusters over the medium-range length scales. The characteristic temperature dependence of the intensity, that is, the rise and fall, confirms the reentrant behaviour.

### Sequence of phase transitions

**Figure 6:**
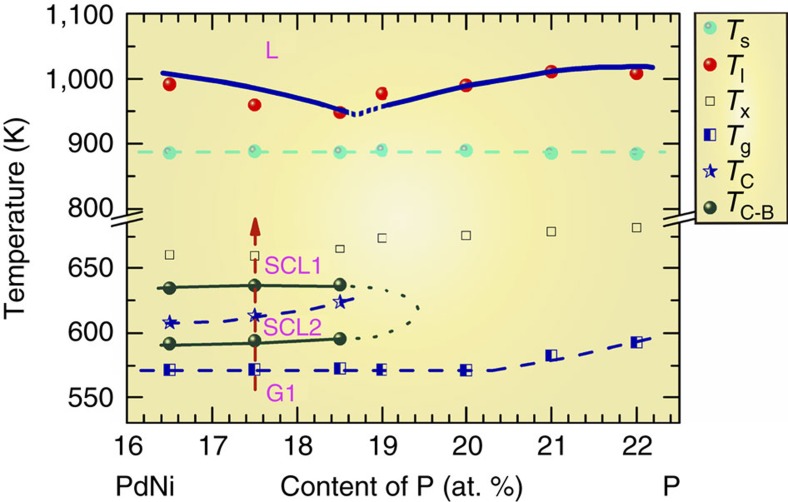
Hidden phase and sequence of phase transitions for (PdNi)_80+*x*_P_20−*x*_ alloys. The diagram is constructed from DSC measurements with a heating rate 20 K min^−1^, for samples prepared with a cooling rate of 5–10^6^ K s^−1^. The arrow indicates the sequence of phase transitions in a typical DSC scan. G1, dynamically arrested glass phase of SCL1; L, liquid phase; SCL1, high temperature supercooled liquid; SCL2, hidden phase in the supercooled liquid; *T*_C_, LLPT temperature; *T*_C−B_, boundaries of the anomalous exothermal peak in DSC; *T*_g_, glass transition temperature; *T*_l_, liquidus temperature; *T*_s_, solidus temperature; *T*_*x*_, crystallization temperature.

A systematic DSC study was completed for a series of Pd-Ni-P samples with varying degrees of P composition. Figure 6 shows a diagram illustrating the hidden phase and the sequence of phase transitions, constructed from characteristic temperatures determined from DSC scans (Supplementary Fig. 3). A sub-*T*_g_ anomaly has been reported by Hu *et al*.^35^, with a strong cooling rate effect in that a hyperquenched glass exhibits a sub-*T*_g_ exothermal peak, whereas a regular glass does not. Although this sub-*T*_g_ exothermal peak is not directly related to our DSC anomaly (which is above *T*_g_), it points out the clear contrast for samples prepared by different cooling rates. We have also studied the effect of cooling rate on AEP, and the results are shown and discussed in Supplementary Fig. 4 and Supplementary Note 2; the influence of cooling rate can be clearly seen. For clarity, we have specifically noted the applicable heating and cooling rates in Fig. 6, to indicate the dynamic nature of the diagram.

Two supercooled liquid phases are identified in Fig. 6. SCL1 is the high-temperature supercooled liquid phase. The room-temperature glass phase (G1) is the dynamically arrested or frozen state of SCL1. The region bounded by the dark turquoise lines is the low-temperature supercooled phase, SCL2, which is usually bypassed in as-cast samples due to rapid quenching. Heating the sample brought the hidden SCL2 phase to light.

## Discussion

**Figure 7:**
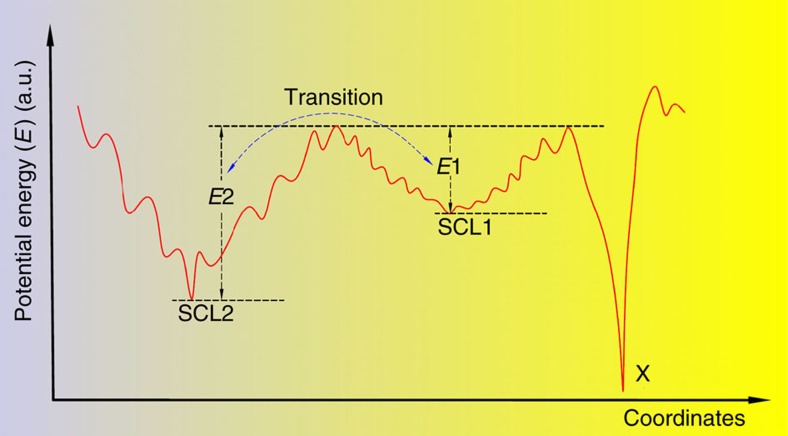
Proposed potential energy landscape in Pd_41.25_Ni_41.25_P_17.5_supercooled liquid. There are three energy minima, marked by SCL1, SCL2 and X, which is a crystalline phase. The shallow, local minima reflect the myriads of atomic configurations. Compared to SCL2, SCL1 is in a more disordered state, with many more configurations.

The energy landscape, a potential energy surface as a function of particle coordinates, offers a convenient way to consider and visualize the structural evolution in the supercooled liquid. Examination of Fig. 6 suggests a potential energy landscape depicted in Fig. 7. There are three deep minima or ‘megabasins' in the potential energy landscape: two supercooled liquid phases (SCL1 and SCL2), and a crystalline phase (X). The crystalline phase has the deepest energy minima, but it is difficult to reach during supercooling due to the slow kinetics in metallic glasses. SCL2 has the lower potential energy of the two supercooled liquid phases. However, quenched samples are always in a frozen, or glass, state of SCL1. Only on heating from room temperature is the system able to sample the potential energy landscape and settle into the lower energy phase of SCL2.

Experimental evidence has shown that when *T*>*T*_C_, the sample reenters into the SCL1 phase. This means that at high temperatures, SCL1, although of higher potential energy, is the thermodynamically stable state. To understand this paradox, it is important to consider the entropy term in the Gibbs free energy.

The Gibbs energy is given by 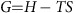, where *H* is the enthalpy or the potential energy and *S* the entropy. Consider the Gibbs energy at a temperature *T*_1_ just below the phase transition (say *T*=570 K) and at *T*_C_. 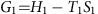 and 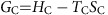. Since SCL1 is the stable phase at *T*_1_, 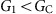. However, 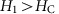, because there is heat release on transforming to SCL2 as evidenced from the *C*_p_ data. Therefore, 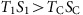. It follows that 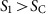, because *T*_1_ is lower. This analysis shows that SCL1 is more disordered, and that SCL1 is stabilized by the entropy term. In Fig. 7, on the potential energy landscape, the more disordered state SCL1 is illustrated with many more local configurations than SCL2. This scenario is analogous to the ordering of a ferromagnet, where the paramagnetic phase at high temperature is stabilized by the entropy of disordered spins^36^.

The presence of ‘megabasins' had been previously discussed by Debenedetti and Stillinger^37^. By comparing molten SiO_2_, a prototypical strong glass former, and *o*-terphenyl, a fragile glass former, they argued that the potential energy landscape of strong glass formers may consist of a single ‘megabasin', whereas the fragile ones display a proliferation of well-separated ‘megabasins.' Pd-Ni-P samples are known to be form a fragile glass^38^. In this regard, the potential energy landscape depicted in Fig. 7 is consistent with the general analysis by Debenedetti and Stillinger^37^.

Structure wise, the phase transitions at *T*_C_ is accomplished through rearrangement of locally favoured structures, or atomic clusters. Figure 5 shows that the cluster rearrangement occurs most prominently at medium-range length scales, at the fifth shell (*r*=11 Å) or longer. Zheng *et al*.^39^ observed three exothermal responses (peaks) in glassy ribbons of Cu_46_Zr_42_Al_7_Y_5_. Based on DSC analysis, they attributed the second peak to the formation of ordered clusters. This work is consistent with an earlier study by Kumar *et al*.^10^, who speculated that the anomalous DSC in Zr_36_Ti_24_Be_40_ could be due to development of short-range order towards the final crystallization product. The high-quality synchrotron data obtained in the present experiment allowed us to visualize the dramatic changes in medium-range order, at the fifth shell (R5) centred around *r*∼11–12 Å.

As stated earlier, the AEP is not unique to Pd-based BMGs; it has been found in a variety of metallic glass systems as well, although the effects are less pronounced and the interpretations vary widely. More interestingly, a systematic study has found a link between the AEP and glass-forming ability (GFA). This was demonstrated for Fe-based BMGs, which have broad applications in electricity delivery due to their exceptional soft magnetic properties. Fe-based BMGs with an AEP tend have better GFA than those without^20^,^21^,^22^,^25^. Other BMGs with an AEP are reported to have improved plasticity^17^,^18^,^19^. Here again, no evidence of nanoscale phase separation was found. Given the similarity, it is likely that the DSC anomalies observed in a variety of BMGs arise from the same structural origin, that is, an LLPT in the supercooled liquid region like the one found in Pd-Ni-P.

The GFA of a metallic glass is influenced by a number of factors^40^,^41^,^42^,^43^,^44^,^45^. It is encouraging that in alloys exhibiting an AEP, there appears to be a correlation between GFA and the AEP. This finding can potentially provide a viable method to tune the GFA. However, additional studies (for example, *ab initio* molecular dynamics simulations^46^) will be required to fully establish the relationship and to clarify the underlying mechanisms.

In conclusion, we reveal a hidden amorphous phase and the associated LLPT in a Pd-Ni-P supercooled liquid. This finding explains four decades long puzzle about the thermodynamic anomaly in DSC scans. At the transition temperature, *T*_C_, the underlying atomic structure undergoes significant changes over the medium-range length scale, as evidenced by *in situ* synchrotron diffraction data. In addition, the SANS data demonstrated the coexistence of two supercooled liquid states in the vicinity of *T*_C_. While the focus of the present study is on Pd-Ni-P alloys, the DSC anomaly has been reported in a number of other BMGs. That the appearance of this DSC anomaly has been linked to the excellent glass-forming ability or GFA in Fe-based BMGs raises a new possibility to control the GFA and properties of BMGs. Because *T*_C_ is rather low, close to *T*_g_ as opposed to the melting temperature, it is conceivable that conventional metallurgical methods, such as heat treatment, could be employed as a means to manipulate the phase and microstructure of the metallic glass of interest.

## Methods

### Sample preparation

A fluxing technique was employed to prepare Pd_41.25_Ni_41.25_P_17.5_ and Pd_40_Ni_40_P_20_ BMG ingots of diameter ∼10 mm with air quenching^1^,^47^. Pd-Ni-P BMG ingots were cut into small pieces of mass ∼43 mg for heat capacity measurements. Heat treatment of Pd_41.25_Ni_41.25_P_17.5_ MGs around *T*_C_ is described in Supplementary Methods. The synchrotron X-ray diffraction results for the heat-treated Pd_41.25_Ni_41.25_P_17.5_ MGs are given in Supplementary Fig. 5.

### Heat capacity measurements

The specific heat capacity curves during heating were measured with reference to a sapphire standard using platinum crucibles with Netzsch DSC 404 F3 on *C*_p_ measurement mode in a high-purity Ar atmosphere.

### *In situ* synchrotron diffraction measurements

Time-resolved high-energy synchrotron X-ray measurements during constant heating were carried out at the beamline 11-ID-C at the Advanced Photon Source, Argonne National Laboratory. BMG plates with ∼2 mm thickness were mounted in a Linkam THMS600 furnace for thermal scanning. High-energy X-rays with a beam size of 0.5 mm × 0.5 mm and wavelength of 0.10804 Å were used in transmission geometry for data collection. The measurement was conducted in a high-purity argon atmosphere. Two-dimensional diffraction patterns were obtained using a Perkin-Elmer amorphous silicon detector. The data acquisition time for each pattern is 1 s. However, including the time for saving data, the total acquisition time is ∼6 s for each pattern. At a constant heating rate of 20 K min^−1^, the temperature precision for each pattern is estimated to be ∼1–2 K. The static structure factor, *S(Q)* with *Q*_max_∼30 Å^−1^, was derived from the scattering data by masking bad pixels, integrating images, subtracting the appropriate background and correcting for oblique incidence, absorption, multiple scattering, fluorescence, Compton scattering and Laue correction^48^ using Fit2D and PDFgetX2. The details of synchrotron data reduction can be found in Supplementary Methods and Supplementary Figs 6 and 7.

### Microscopy analysis

The double Cs-corrected TEM Titan3 G2 60–300 at Hokkaido University was employed to acquire high-resolution TEM images shown in Fig. 3c for a Pd_41.25_Ni_41.25_P_17.5_ BMG air quenched from ∼*T*_C_ in SCL2. The high-voltage electron microscopy JEM-ARM 1300 with an accelerating voltage of 1,250 kV at Hokkaido University was used to acquire high-resolution TEM images and the SAED pattern for a Pd_41.25_Ni_41.25_P_17.5_ BMG air quenched from ∼643 K in SCL1.

### Time-resolved SANS-DSC measurements

Simultaneous SANS-DSC studies during constant heating with a rate 2.5 K min^−1^ were performed on the QUOKKA SANS instrument at the OPAL reactor, Australian Nuclear Science and Technology Organization (ANSTO)^49^. A neutron beam with diameter of 5 mm and wavelength, *λ*, of 5 Å (10% resolution) was used. A DSC and Al crucibles designed by QUOKKA instrument group^50^ were employed. The BMG plates with ∼0.9 mm thickness and with ∼200 mg mass were used for simultaneous SANS-DSC studies. Three configurations were employed for room-temperature measurements covering a *Q* range 0.003–0.727 Å^−1^, where *Q* is the magnitude of the scattering vector defined as 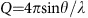 and 2*θ* is the scattering angle. The configuration for *in situ* SANS-DSC study covers a *Q* range 0.0065–0.1 Å^−1^. SANS data were reduced with data corrected for empty cell scattering, transmission and detector response and transformed onto an absolute scale by the use of an attenuated direct beam transmission measurement.

### Data availability

The data that support the findings of this study are available from the corresponding author on request.

## Additional information

**How to cite this article:** Lan, S. *et al*. Hidden amorphous phase and reentrant supercooled liquid in Pd-Ni-P metallic glasses. *Nat. Commun.* **8,** 14679 doi: 10.1038/ncomms14679 (2017).

**Publisher's note**: Springer Nature remains neutral with regard to jurisdictional claims in published maps and institutional affiliations.

## Supplementary Material

Supplementary Information Supplementary Figures, Supplementary Notes, Supplementary Methods and Supplementary References

## References

[b1] Kui H. W., Greer A. L. & Turnbull D. Formation of bulk metallic-glass by fluxing. Appl. Phys. Lett. 45, 615–616 (1984).

[b2] Willnecker R., Wittmann K. & Görler G. Undercooling investigations and heat capacity measurements on Pd-Ni-P melts. J. Non-Cryst. Solids 156, 450–454 (1993).

[b3] He Y., Schwarz R. B. & Archuleta J. I. Bulk glass formation in the Pd-Ni-P system. Appl. Phys. Lett. 69, 1861–1863 (1996).

[b4] Chen H. Glass temperature, formation and stability of Fe, Co, Ni, Pd and Pt based glasses. Mater. Sci. Eng. 23, 151–154 (1976).

[b5] Yavari A., Osamura K., Okuda H. & Amemia Y. Small-angle x-ray scattering study of phase separation in amorphous alloys during heating with use of synchrotron radiation. Phys. Rev. B 37, 7759 (1988). 10.1103/physrevb.37.7759 9944077

[b6] Oehring M. Chemical homogeneity of Nipdp metallic glasses studied by atom probe-field ion microscopy. Z. Metallkd. 80, 1–8 (1989).

[b7] Madge S., Rösner H. & Wilde G. Transformations in supercooled Pd_40.5_Ni_40.5_P_19_. Scr. Mater. 53, 1147–1151 (2005).

[b8] Yavari A., Hamar-Thibault S. & Sinning H. On the microstructure of amorphous Pd_46_Ni_36_P_18_ with two glass transitions. Scr. Metall. 22, 1231–1234 (1988).

[b9] Tanner L. E. & Ray R. Phase separation in Zr-Ti-Be metallic glasses. Scr. Metall. 14, 657–662 (1980).

[b10] Kumar G., Nagahama D., Ohnuma M., Ohkubo T. & Hono K. Structural evolution in the supercooled liquid of Zr_36_Ti_24_Be_40_ metallic glass. Scr. Mater. 54, 801–805 (2006).

[b11] Park E., Chang H. & Kim D. Effect of addition of Be on glass-forming ability, plasticity and structural change in Cu-Zr bulk metallic glasses. Acta Mater. 56, 3120–3131 (2008).

[b12] Hasegawa R. & Tanner L. Superconducting transition temperatures of glassy and partially crystalline Be-Nb-Zr alloys. J. Appl. Phys. 49, 1196–1199 (1978).

[b13] Hays C., Kim C. & Johnson W. Large supercooled liquid region and phase separation in the Zr-Ti-Ni-Cu-Be bulk metallic glasses. Appl. Phys. Lett. 75, 1089–1091 (1999).

[b14] Gao Y. L. . Nanocrystallization of Zr-Ti-Cu-Ni-Be bulk metallic glass. Mater. Lett. 57, 2341–2347 (2003).

[b15] Sohn S., Yook W., Kim W. & Kim D. Phase separation in bulk-type Gd-Zr-Al-Ni metallic glass. Intermetallics 23, 57–62 (2012).

[b16] Na J., Sohn S., Kim W. & Kim D. Two-step-like anomalous glass transition behavior in Ni-Zr-Nb-Al-Ta metallic glass alloys. Scr. Mater. 57, 225–228 (2007).

[b17] Xu D., Duan G. & Johnson W. L. Unusual glass-forming ability of bulk amorphous alloys based on ordinary metal copper. Phys. Rev. Lett. 92, 245504 (2004). 15245096 10.1103/PhysRevLett.92.245504

[b18] Park E., Lee J. & Kim D. Effect of Ag addition on the improvement of glass-forming ability and plasticity of Mg-Cu-Gd bulk metallic glass. J. Mater. Res. 20, 2379–2385 (2005).

[b19] Park E., Na J. & Kim D. Abnormal behavior of supercooled liquid region in bulk-forming metallic glasses. J. Appl. Phys. 108, 053515 (2010).

[b20] Huang X., Wang X., He Y., Cao Q. & Jiang J. Are there two glass transitions in Fe-M-Y-B (M=Mo, W, Nb) bulk metallic glasses? Scr. Mater. 60, 152–155 (2009).

[b21] Lee S. . Excellent thermal stability and bulk glass forming ability of Fe-B-Nb-Y soft magnetic metallic glass. Mater. Trans. 49, 506–512 (2008).

[b22] Han Z., Zhang J. & Li Y. Quaternary Fe-based bulk metallic glasses with a diameter of 5mm. Intermetallics 15, 1447–1452 (2007).

[b23] Zhang W. . Two-stage-like glass transition and the glass-forming ability of a soft magnetic Fe-based glassy alloy. J. Appl. Phys. 105, 3518 (2009).

[b24] Li J. W., Men H. & Shen B. L. Soft-ferromagnetic bulk glassy alloys with large magnetostriction and high glass-forming ability. AIP Adv. 1, 343–347 (2011).

[b25] Li J. W. . Thermal stability, magnetic and mechanical properties of Fe-Dy-B-Nb bulk metallic glasses with high glass-forming ability. Intermetallics 46, 85–90 (2014).

[b26] Martin I., Ohkubo T., Ohnuma M., Deconihout B. & Hono K. Nanocrystallization of Zr_41.2_Ti_13.8_Cu_12.5_Ni_10.0_Be_22.5_ metallic glass. Acta Mater. 52, 4427–4435 (2004).

[b27] Nagahama D., Ohkubo T. & Hono K. Crystallization of Ti_36_Zr_24_Be_40_ metallic glass. Scr. Mater. 49, 729–734 (2003).

[b28] Pham K. N. . Multiple glassy states in a simple model system. Science 296, 104–106 (2002). 11935020 10.1126/science.1068238

[b29] Shimizu R., Kobayashi M. & Tanaka H. Evidence of liquid–liquid transition in triphenyl phosphite from time-resolved light scattering experiments. Phys. Rev. Lett. 112, 125702 (2014). 24724660 10.1103/PhysRevLett.112.125702

[b30] Liu L., Chen S. H., Faraone A., Yen C. W. & Mou C. Y. Pressure dependence of fragile-to-strong transition and a possible second critical point in supercooled confined water. Phys. Rev. Lett. 95, 117802 (2005). 16197049 10.1103/PhysRevLett.95.117802

[b31] Wei S. . Liquid–liquid transition in a strong bulk metallic glass-forming liquid. Nat. Commun. 4, 2083 (2013). 23817404 10.1038/ncomms3083

[b32] Xu W. . Evidence of liquid–liquid transition in glass-forming La_50_Al_35_Ni_15_ melt above liquidus temperature. Nat. Commun. 6, 7696 (2015). 26165855 10.1038/ncomms8696 PMC4510689

[b33] Lan S. . Structural crossover in a supercooled metallic liquid and the link to a liquid-to-liquid phase transition. Appl. Phys. Lett. 108, 211907 (2016).

[b34] Sheng H. W. . Polyamorphism in a metallic glass. Nat. Mater. 6, 192–197 (2007). 17310140 10.1038/nmat1839

[b35] Hu L., Zhou C., Zhang C. & Yue Y. Thermodynamic anomaly of the sub-T(g) relaxation in hyperquenched metallic glasses. J. Chem. Phys. 138, 174508 (2013). 23656145 10.1063/1.4803136

[b36] Bao W. . Unconventional ferromagnetic and spin-glass states of the reentrant spin glass Fe_0.7_Al_0.3_. Phys. Rev. Lett. 82, 4711–4714 (1999).

[b37] Debenedetti P. G. & Stillinger F. H. Supercooled liquids and the glass transition. Nature 410, 259–267 (2001). 11258381 10.1038/35065704

[b38] Tanaka H. Relation between thermodynamics and kinetics of glass-forming liquids. Phys. Rev. Lett. 90, 055701 (2003). 12633377 10.1103/PhysRevLett.90.055701

[b39] Zheng H. . Thermodynamic evidence for cluster ordering in Cu_46_Zr_42_Al_7_Y_5_ ribbons during glass transition. Sci. Bull. 61, 706–713 (2016).

[b40] Turnbull D. Under what conditions can a glass be formed? Contemp. Phys. 10, 473–488 (1969).

[b41] Lu Z. P. & Liu C. T. Glass formation criterion for various glass-forming systems. Phys. Rev. Lett. 91, 115505 (2003). 14525439 10.1103/PhysRevLett.91.115505

[b42] Laws K. J., Miracle D. B. & Ferry M. A predictive structural model for bulk metallic glasses. Nat. Commun. 6, 8123 (2015). 26370667 10.1038/ncomms9123 PMC4648055

[b43] Johnson W. L., Na J. H. & Demetriou M. D. Quantifying the origin of metallic glass formation. Nat. Commun. 7, 10313 (2016). 26786966 10.1038/ncomms10313 PMC4735709

[b44] Inoue A. Stabilization of metallic supercooled liquid and bulk amorphous alloys. Acta Mater. 48, 279–306 (2000).

[b45] Greer A. L. Materials science—confusion by design. Nature 366, 303–304 (1993).

[b46] Guan P. F., Fujita T., Hirata A., Liu Y. H. & Chen M. W. Structural origins of the excellent glass forming ability of Pd_40_Ni_40_P_20_. Phys. Rev. Lett. 108, 175501 (2012). 22680882 10.1103/PhysRevLett.108.175501

[b47] Nishiyama N. & Inoue A. Supercooling investigation and critical cooling rate for glass formation in Pd-Cu-Ni-P alloy. Acta Mater. 47, 1487–1495 (1999).

[b48] Egami T., Dmowski W., He Y. & Schwarz R. Structure of bulk amorphous Pd-Ni-P Alloys determined by synchrotron radiation. Metall. Mater. Trans. A 29A, 1805–1809 (1998).

[b49] Gilbert E. P., Schulz J. C. & Noakes T. J. ‘Quokka'- the small-angle neutron scattering instrument at OPAL. Phys. B 385-86, 1180–1182 (2006).

[b50] Pullen S. A. . Design and implementation of a differential scanning calorimeter for the simultaneous measurement of small angle neutron scattering. Meas. Sci. Technol. 25, 055606 (2014).

