# Supplementary material for: Hidden amorphous phase and reentrant supercooled liquid in Pd-Ni-P metallic glasses
Source: Nat Commun. 2017 Mar 17;8:14679. doi: 10.1038/ncomms14679 (PMC5357859; doi:10.1038/ncomms14679)
Supplement: Supplementary Information — Supplementary Figures, Supplementary Notes, Supplementary Methods and Supplementary References [file ncomms14679-s1.pdf]

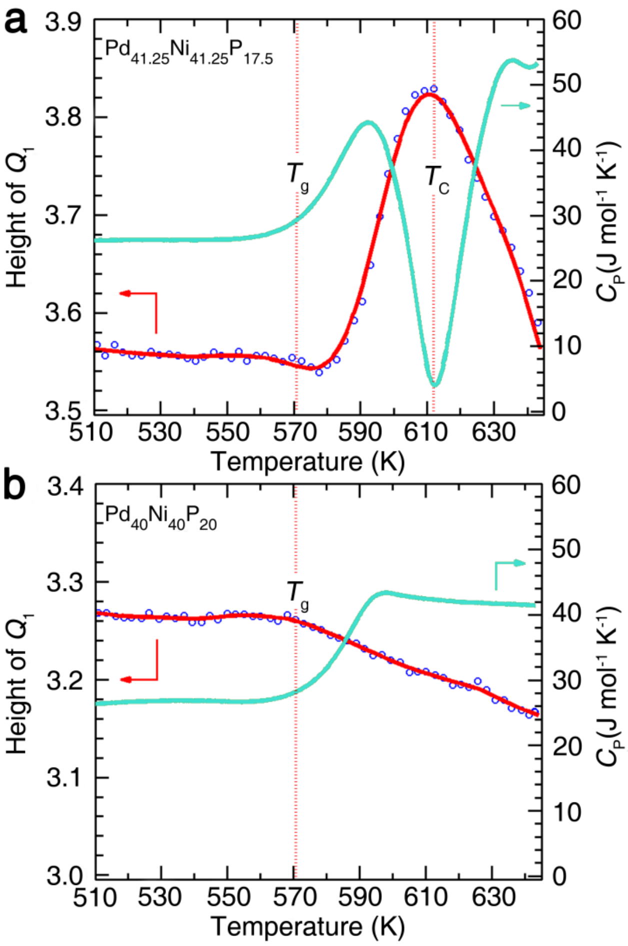

Supplementary Figure 1. Temperature dependence of the first sharp diffraction peak Q_1.

The peak height of Q_1 is compared with C_p for **(a)** $Pd_{41.25}Ni_{41.25}P_{17.5}$ and **(b)** $Pd_{40}Ni_{40}P_{20}$ BMGs. The T_g and T_C are marked by vertical dashed lines. The red solid lines are spline fits for the peak height data.

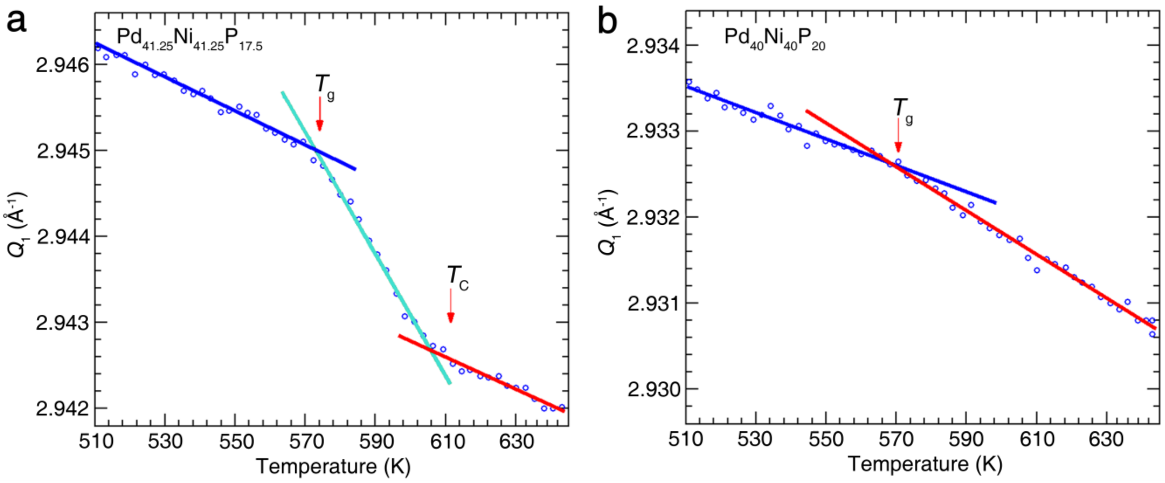

Supplementary Figure 2. Changes of the peak position of Q_1 upon heating. The peak position of Q_1 as a function of temperature for **(a)** $Pd_{41.25}Ni_{41.25}P_{17.5}$ and **(b)** $Pd_{40}Ni_{40}P_{20}$ BMGs. The solid lines are the results of linear fits for different temperature regimes. The first moment represents the peak position, which was calculated by the formula $Q_1 = \sum S(Q) \cdot Q / \sum S(Q)$. The slope changes at T_g and T_C indicates the occurrence of transitions.

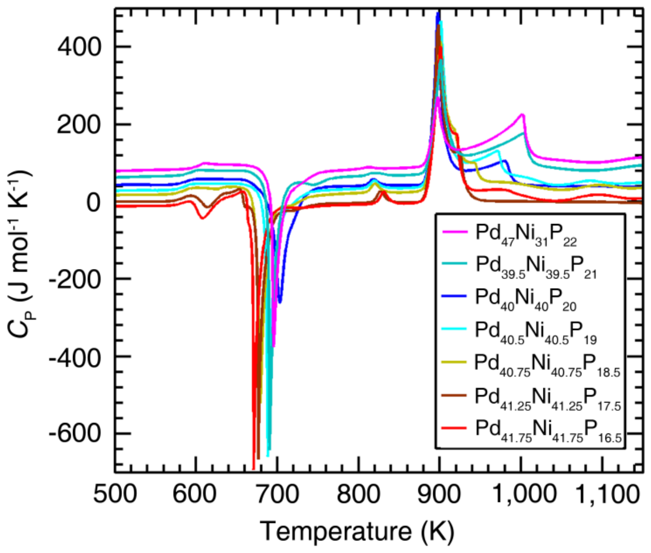

Supplementary Figure 3. DSC data during heating for Pd-Ni-P metallic glasses. The heating rate is 20 K min^{-1}. The curves have been shifted vertically for clarity. Characteristic temperatures T_g, T_C, T_x, T_s and T_l, were determined from these data.

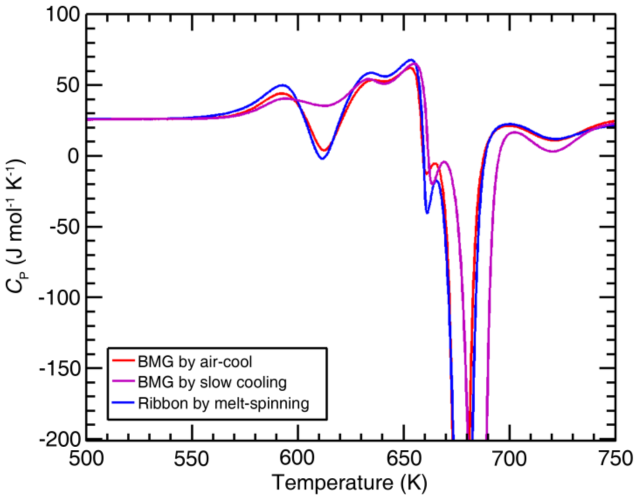

Supplementary Figure 4. Effect of cooling rates on DSC anomaly. Cp data for $Pd_{41.25}Ni_{41.25}P_{17.5}$ metallic glasses at different cooling rates. The cooling rates for air-cooled, and slow cooled as well as the melt-spinning samples were estimated to be ~ 5 K s^{-1}, ~ 2 K s^{-1} and ~10^6 K s^{-1}, respectively.

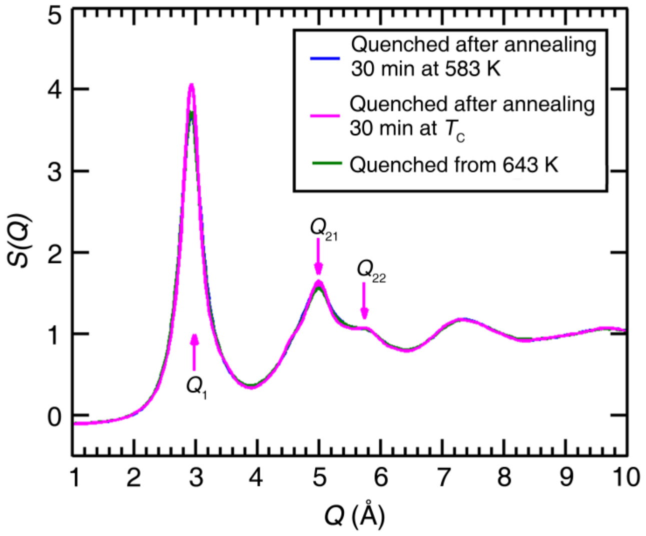

Supplementary Figure 5. Change of structure factors upon heat-treatment around T_C.

The X-ray structure factors *S(Q)* are compared for $Pd_{41.25}Ni_{41.25}P_{17.5}$ metallic glasses quenched after isothermal annealing for 30 min at 583 K (close to T_g), at T_C, and at 643 K (~ 32 K above T_C).

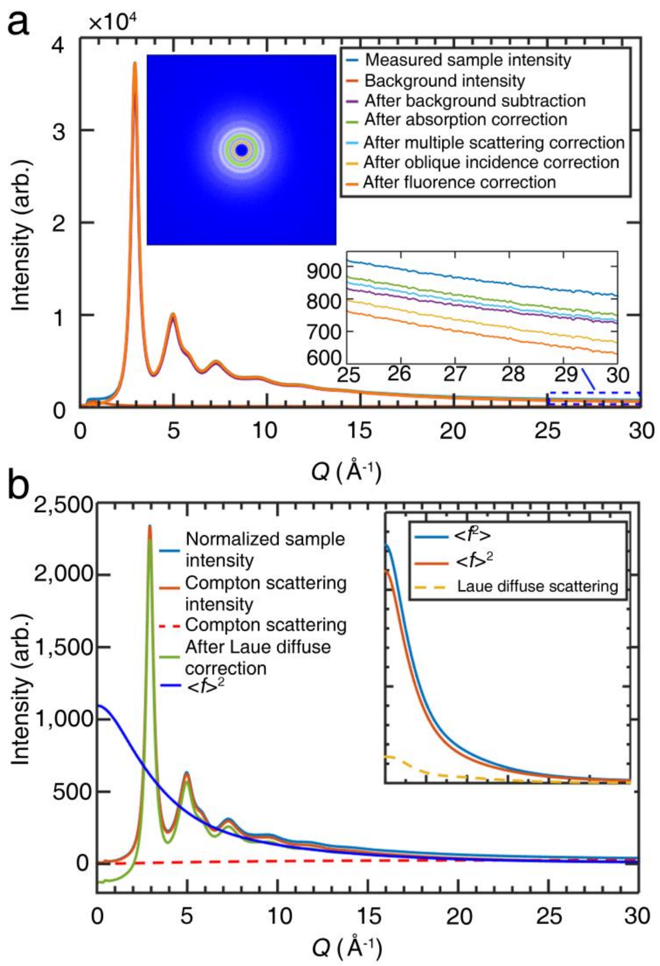

Supplementary Figure 6. Step-by-step corrections to obtain the *S(Q)*. (a) Background subtraction, absorption, multiple scattering, oblique incidence, fluorescence corrections etc. **(b)** Normalization, Compton scattering, and Laue diffuse scattering corrections.

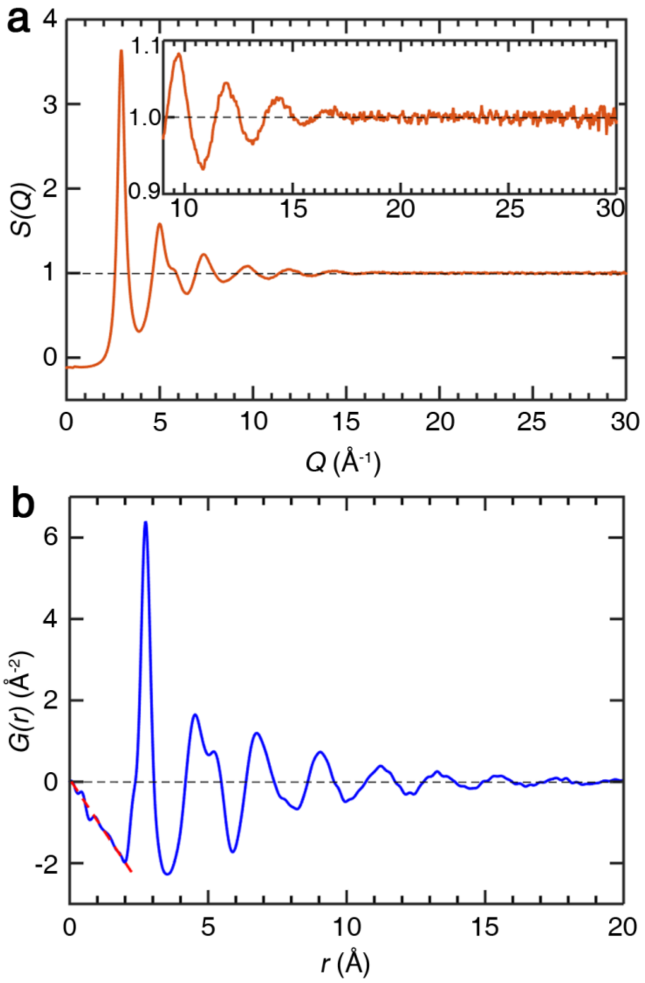

Supplementary Figure 7. Representative reduced diffraction data in reciprocal (Q) and real space (r). (a) *S(Q)* and **(b)** *G(r)*.

Supplementary Note 1: Slope changes of peak position Q_1 at T_g and T_c

It can be seen in Supplementary Figure 2 that at low temperatures, the position of Q_1 decreases with increasing temperature for both samples. The slope changes at T_g, indicating a structure cross-over. In $Pd_{41.25}Ni_{41.25}P_{17.5}$, however, another transition is seen at T_C. Moreover, above T_C (and below T_x), the slope resumes its value for $T< T_g$. This result once again confirms the reentrant behavior for $Pd_{41.25}Ni_{41.25}P_{17.5}$.

Supplementary Note 2: Sample preparation for Pd-Ni-P BMGs with different cooling rates

Pd-Ni-P bulk metallic glasses (BMGs) with different thermal history were prepared to study the effect the cooling rates. The air-cooled BMG was prepared using the fluxing technique as mentioned in the experiment section of the main text. The total time for cooling the molten liquid (~ 1473 K) to the glass transition temperature (~ 573 K) in air was recorded to be ~ 180 s, yielding an average cooling rate of ~ 5 K s^{-1}.

Slow-cooled samples were prepared by cooling the alloys coated with B_2O_3 flux with a quartz tube in a furnace with a preset temperature ~ 568 K (5 K below the T_g in the DSC curve using 20 K min^{-1} heating rate). The recorded cooling time from high temperature (~ 1473) to the preset furnace temperature (~ 568 K) is ~ 300 s, which corresponds to an average cooling rate ~ 2 K s^{-1}. As shown in Supplementary Figure 4, the DSC measurement for the $Pd_{41.25}Ni_{41.25}P_{17.5}$ metallic glass with a slower cooling rate exhibits a shallower exothermic peak at T_C ~ 612 K compared to that of the air-cooled sample.

Glassy ribbon samples were also prepared to illustrate the influence of fast cooling rate on the anomalous exothermic peak above T_g. Ingots of alloys were prepared by fluxing techniques first and then ribbons were prepared using melt-spinning. The speed of melt-spinning was ~ 30 m s^{-1} corresponding to a cooling rate ~ 10^6 K s^{-1}. As shown in Supplementary Figure 4, a DSC measurement at a 20 K min^{-1} heating rate confirmed that there is a similar exothermic peak at T_C ~ 612 K.

Supplementary Methods

Heat-treatment of $Pd_{41.25}Ni_{41.25}P_{17.5}$ MGs around T_C and the related synchrotron X-ray diffraction measurements

The metallic glasses were first prepared by air-cooling. The heat-treated samples were prepared by heating up the as-cast metallic glasses to the appropriate temperatures in sealed high-vacuum quartz tubes and then quenched in the flowing water to freeze the microstructure. The structure of as-prepared metallic glasses was examined using high-energy synchrotron X-ray diffraction with the same parameters as shown in the main text at 11-ID-C of APS. The structure factors, $S(Q)$, for samples quenched from 583 K and 643 K are similar. On the other hand, the $S(Q)$ for the metallic glass sample annealed at T_C exhibits more features. As shown in Supplementary Figure 5, The Q_1, Q_{21}, and Q_{22} became sharper, which is consistent with the results shown in the main text. High Resolution TEM (HRTEM) was also used to confirm the amorphous nature of the as-prepared metallic glasses

(not shown here). The above features for samples with different thermal histories illustrated that the structure of the metallic glasses could be controlled using heat-treatment around T_C.

Data reduction procedure for *S(Q)*

The basic theory for obtaining *S(Q)* from synchrotron or neutron scattering has been given by Billinge and Egami [1]. In practice, *S(Q)* was reduced using software PDFgetX2 [2,3]. The incident beam flux was normalized using the ion-chamber counts.

(a) Raw data reduction and polarization correction in Fit2D

The raw data are 2-D images collected using a GE detector plate, as shown by the inset in Supplementary Figure 6. Fit2D was employed for Φ integration of the diffraction pattern images, where Φ is the azimuthal angle of the scattering vector. A polarization correction factor is also applied in this step.

(b) Intensity Correction

The background intensity mainly comes from the environmental (e.g., air) scattering, which has been subtracted at the beginning of the intensity corrections. The sample scattering intensity, I_{samp}, of the as-cast $Pd_{41.25}Ni_{41.25}P_{17.5}$ bulk metallic glass can therefore be expressed as

$$I_{\mathrm{samp}} = \alpha(I_{\mathrm{corr}} + I_{\mathrm{multiple}} + I_{\mathrm{Fluor}}) \qquad (1)$$

where,

I_{samp} - the sample intensity after background subtraction; α - the absorption factor, which depends on the sample geometry and attenuation coefficients of relevant elements; I_{multiple} - the multiple scattering contribution; I_{Fluor} - the isotropic fluorescence correction.

I_{corr} is obtained after corrections outlined in Eq. (1). Step-by-step corrections, including absorption correction, multiple scattering (two orders only) correction, and oblique incidence correction (for flat detector plate), as well as fluorescence correction, were applied in sequence and illustrated in Supplementary Figure 6.

(c) Normalization, correction for Compton scattering

The measured scattering intensity is then corrected for the X-ray scattering form factor, calculated from tabulated values. By integrating over a finite and selected range of the high-q data, we can calculate the overall normalization factor N in equation (2). Compton scattering correction was applied to reduce the incoherent scattering. The tabulated data were used to describe the Compton scattering profile in PDFgetX2.

$$I_{\mathrm{corr}} = N(I_{\mathrm{coh}} + I_{\mathrm{incoh}}) \qquad (2)$$

where,

N - a normalization factor; I_{coh} - the coherent, elastic scattering contribution; I_{incoh} - the incoherent or Compton scattering contribution.

(d) Laue diffuse scattering and weighting factor

Laue diffuse scattering correction accounts for scattering when there is more than one atom-type in the sample, which is generally given by ($<f^2>-<f>^2$) and decreases monotonically with increasing momentum transfer Q. As ref. 1 shows, the structure factor *S(Q)* is defined as:

$$S(Q) = \frac{I_{\mathrm{coh}} - (<f^2> - <f>^2)}{<f>^2} \qquad (3)$$

The X-ray form factors $<f^2>$ and $<f>^2$ are calculated using tabulated values. Thus, at high Q, the coherent scattering after Laue diffuse scattering correction oscillates around $<f>^2$. The weighing factor is set to $<f>^2$ in PDFgetX2 to obtain *S(Q)* [1,2]. Step-by-step correction to obtain the X-ray structure factor *S(Q)* is shown in Supplementary Figure 6. The reduced *S(Q)* and *G(r)* are shown in Supplementary Figure 7.

Supplementary References

1. Egami, T., and Billinge, S., Underneath the Bragg peaks: Structure Analysis of Complex Materials, Pergamon, (2003).

2. Qiu, X., Tompson, J. W., Billinge, S. J. L., PDFgetX2: A GUI driven program to obtain the pair distribution function from X-ray diffraction data, J. Appl. Cryst. **37**, 678-678 (2004).

3. Qiu, X., Tompson, J. W., Billinge, S. J. L., PDFgetX2, http://www.pa.msu.edu/cmp/billinge-group/programs/PDFgetX2/, 2009.
